# Viruses, bacteria, vectors, and climate change: how worried should the Americas be?

**DOI:** 10.1016/j.lana.2024.100675

**Published:** 2024-01-15

**Authors:** The Lancet Regional Health – Americas

Climate change threatens life in many dimensions, from the most direct examples, such as extended droughts affecting food production and water supply and heat-related deaths, to the less obvious and indirect, like labour capacity loss and changes in the epidemiology of vector-borne diseases. Data from the World Bank projects that 21 million people could die as a result of a warmer climate by 2050 from just five health risks: extreme heat, stunting, diarrhoea, malaria, and dengue; the last two a direct consequence of changes in vector distribution in response to climate change. We have already started to see the effects, with the expansion of dengue to temperate climates in Europe, the USA, and South America in a stark reminder that climate change is not just about melting ice caps and rising sea levels—it's about the real, tangible impact on the lives of everyday people.

July, 2023 was the hottest month ever recorded and in the past year, heat records were broken in all continents. As temperatures increase and change landscapes, climates, and biomes, the epidemiology of vector-borne diseases also changes, with new threats emerging and old ones becoming more frequent. With milder winters and longer and hotter spring and summer, the USA experienced the worst tick season this far. Once confined to certain areas and predictable periods, many tick species have expanded their presence to northern US states and urban areas. Ticks can transmit deadly diseases to humans and animals. The black-legged tick (or deer tick) can carry *Borrelia burgdorferi*, the bacteria that causes Lyme disease. Black-legged ticks used to be found mainly in the northeast USA but are now common all the way up to eastern Canada. The Gulf Coast tick that can carry *Rickettsia parkeri* and cause rickettsiosis—a type of spotted fever, was once called a southeaster tick but is now established in New Jersey. In a recent outbreak, 5 people in California died of Rocky Mountain spotted fever (RMSF), transmitted by brown dog ticks, after visiting the same town in northern Mexico. RMSF is endemic in northern Mexico and, by contrast with the usual association with outdoor activities for Lyme disease, RMSF has established in densely populated urban areas, challenging the suspected diagnosis as we used to know. The epidemiology of tick-borne diseases is changing together with the climate, threatening new populations and forcing health-care workers to adapt. The recent outbreak in California is a clear warning. The earlier we suspect tick-borne diseases, the higher the chances of treatment and saving lives.

The rising temperatures and erratic precipitation patterns fuelled by climate change have become the perfect breeding grounds for disease-carrying vectors like ticks and mosquitoes. The expansion of *Aedes* mosquitoes, which are vectors for dengue, chikungunya, and zika viruses, threatens to expose naive populations and can potentially generate new epidemic waves like those observed in Central and South America in 2013–2018. As a compounding factor, mosquito-borne arbovirus infections are intrinsically associated with the most vulnerable and minoritised populations who live in poor, densely populated areas and with limited access to sanitation. As one of the most unequal regions of the world, South America is likely to experience the most devastating consequences of such expansion of arboviral diseases and, once again, those who often contributed least to climate change will be disproportionately affected.

After decades of research, some concrete advances in arbovirus vaccines were seen in the past year, bringing some much-needed hope. The US Food and Drug Administration recently approved Ixchiq, the first vaccine for chikungunya virus, and in response to the surge in cases, the WHO Strategic Advisory Group of Experts on Immunization shared recommendations for the use of a new dengue tetravalent vaccine (QDENGA/TAK-003, Takeda) in high-burden settings. The only dengue vaccine licensed in the USA (Dengvaxia/CYD-TDV, Sanofi) is effective only for those previously infected with the virus, being useless for the unexposed population at risk in the country, but TAK-003 is protective for both exposed and unexposed groups. Both dengue vaccines have been approved in Brazil, which has the highest-burden areas for dengue, and on Dec 22, 2023, the Brazilian Minister of Health announced that TAK-003 will be incorporated into the National Vaccination Program, making the country the first one to offer dengue vaccine for free through the healthcare system from February, 2024. However, limited manufacturing capacity will limit the access to priority groups defined based on risk exposure. Limited access to vaccines is likely to be the greatest barrier for all countries in the region and will be most harmful to those without universal health access, with the highest impact on those who need the most.

Although the development of vaccines is a formidable advance and will be an essential tool to prevent the spread and reduce the burden of disease, we must not lose sight of the bigger issue, the intricate link between a changing climate and the surge of infectious diseases. The consequences of our actions—and inactions—are showing. The warmer climate paired with deforestation, irrational land use, and the extinction of natural hosts fuel the increase of vector-borne diseases. A unified, robust strategy to combat vector-borne diseases must reflect the interconnectedness of environmental and human health, making a One Health approach the most powerful strategy moving forward, and leaders must recognise that the clock is ticking.

In 2023, the 28th UN Climate Change Conference (COP 28) dedicated, for the first time, an entire day to discuss the effects of climate change on health. Although this is a commendable advance and a step in the right direction, any mitigation or adaptation discussion that does not include a radical compromise to curb carbon emissions is designed to fail. The reluctance to adopt stringent measures to curb carbon emissions directly contributes to the changing climate conditions that foster the expansion of disease-carrying vectors and exacerbates the vulnerability of communities already on the front lines of climate change.

The time for empty promises and symbolic gestures has long passed. In the absence of decisive action, we risk witnessing a public health crisis of unprecedented proportions, with devastating social and economic ramifications. We should all be worried and demand more than diplomatic niceties. It is time for a commitment to change, with policies that prioritise the wellbeing of people over political expediency to give the American people and the rest of the world a glimpse of hope.

